# A tale of two tissues: Probing gene expression in a complex insect‐induced gall

**DOI:** 10.1111/mec.16482

**Published:** 2022-05-06

**Authors:** Jack C. Schultz, Graham N. Stone

**Affiliations:** ^1^ Department of Environmental Sciences University of Toledo Toledo Ohio USA; ^2^ Institute of Evolutionary Biology University of Edinburgh Edinburgh UK

**Keywords:** Cynipidae, *Dryocosmus quercuspalustris*, gall wasp, gene expression, oak, plant gall

## Abstract

Plant galls are novel and sometimes dramatic plant organs whose development is initiated and controlled by parasitic microbes, nematodes, insects and mites. For arthropods, galls provide relative safety from enemies and abiotic stresses while providing nutrition. Galls are formed entirely by the plant, whose transcriptional pathways are modified and coopted to produce a structure specific to the galler species; they comprise a classic example of Dawkins’ “extended phenotype”. Arthropod‐elicited galls are unique in that they are often anatomically complex (Figure 1a), with multiple differentiated tissue types (Figure 1b). A growing number of investigators have studied changes in hostplant gene expression to understand arthropod gall development. In this issue of *Molecular Ecology*, Martinson et al. (2021) report using RNA sequencing to explore tissue‐specific gene expression associated with anatomical and functional gall complexity, demonstrating for the first time that gall tissues are as different transcriptionally as they are anatomically.

Transcriptional studies have often found changes in the expression of thousands of genes during gall development (e.g., Betancourt et al., 2020; Hearn et al., 2019; Nabity et al., 2013; Schultz et al., 2019). But these studies have sampled entire galls, which allows comparison of gall and nongall plant tissues but provides no insight into tissue‐specific transcription underlying gall complexity. Martinson et al. (2021) have for the first time quantified tissue‐specific gene expression in a gall induced by a gallwasp (Hymenoptera: Cynipidae), *Dryocosmus quercuspalustris* (Osten Sacken, 1861) on leaves of northern red oak (*Quercus rubra* L.) (Figure 1a,b).

**FIGURE 1:**
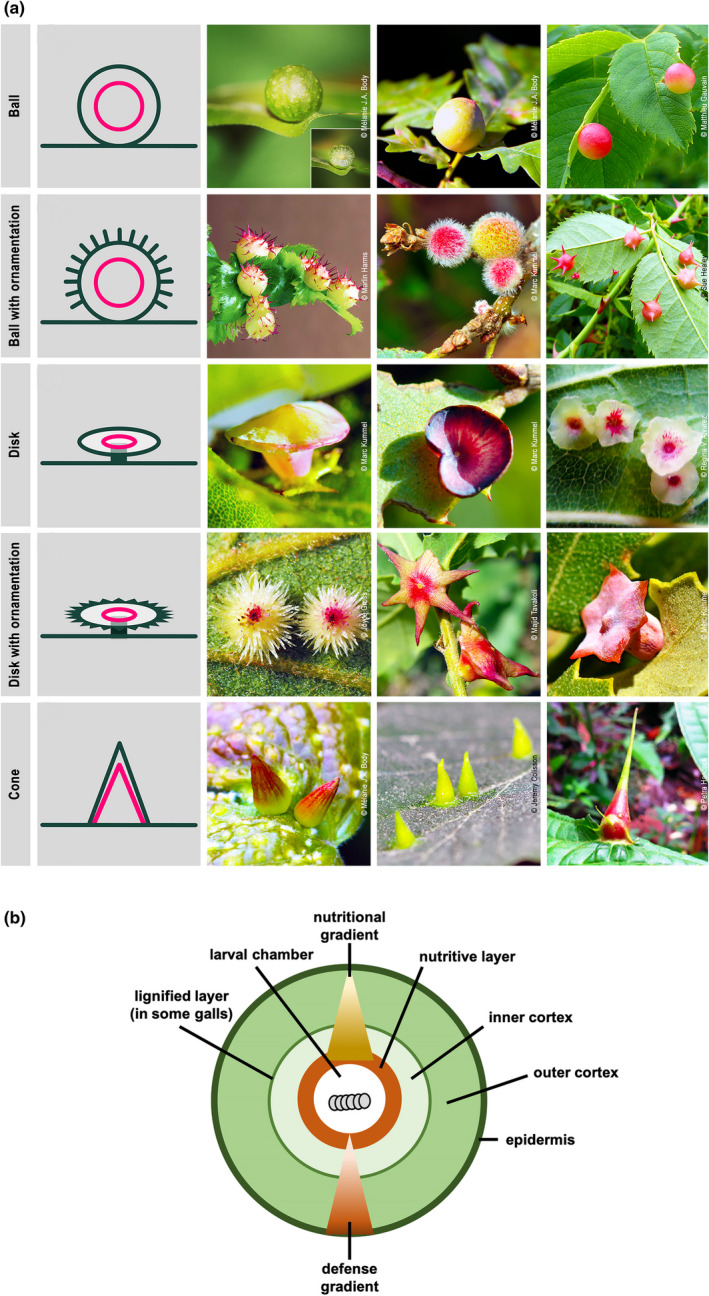
(a) Examples of insect gall complexity, featuring Cynipidae (gall wasps). Red lines in the diagram illustrate locations of nutritive layers, on which the insect feeds. Each example of a shape is elicited by a different insect species. Photo credits: Marc Kummel, Melanie J.A. Body, Joyce Gross, Matthieu Gauvain, Sue Healey, Regina V. Alvarez, Majid Tavakoli, Jeremy Collison, and Petra Hancock all with permission. (b) Simplified, generalized diagram of a gall wasp gall. Triangles illustrate concentration of nutrients near the insect, and concentration of defences away from it

Like many cynipid galls, the globular *D*. *quercuspalustris* gall is anatomically complex. Elicited by egg and fluid injected into very young leaves in the bud, its structure is unlike any normal oak organ. The gall interior is clearly divided into several major tissue types. The wasp larva develops in an inner capsule lined with cells on which it feeds, and which is suspended in the hollow outer gall cortex by connecting filaments.

Martinson et al. (2021) demonstrate that this gall is complex transcriptionally as well as anatomically. They assembled a de novo transcriptome for *Q*. *rubra* from RNA extracted from inner and outer gall and adjacent leaf tissues, then identified genes whose expression differed significantly among them. They found large gene expression changes in the gall, with 28% of oak genes differentially expressed in the gall versus leaf. What is unique in this study is the discovery of similar gene expression differences within a gall.

If the gall is different from a leaf, then what is it? A principal components analysis incorporating additional transcriptional data from other studies found that the outer gall transcriptome resembled that from twigs, leaf buds and reproductive structures but not leaves. In contrast, the inner gall transcriptome was unlike that of any normal oak tissues. Martinson et al. (2021) sampled ungalled leaf tissue close to the attachment point for the developing gall as ungalled control. Differences between gall and leaf samples would probably be even greater had control samples been taken farther from the gall. Nonetheless it is clear that the leaf cells from which the gall arose underwent a dramatic change in developmental trajectory and identity to create a unique and novel organ.

Where do resources for the developing gallwasp come from? Although outer gall tissues are green, Martinson et al. (2021) found expression of photosynthesis‐related genes to be reduced in outer gall tissues relative to ungalled leaves, and suppressed or blocked in inner tissues, suggesting that inner gall tissues are effectively heterotrophic. This is compatible with a widespread view that galls are sinks that draw plant metabolites and nutrients from surrounding nongall tissues (Giron et al., 2016), and the few galling species known to negatively impact hostplant fitness may do so by outcompeting naturally occurring sinks (e.g., galling aphids on poplar and Hessian Fly on wheat, Compson et al., 2011; Subramanyam et al., 2021). Elevated expression of genes related to sugar and amino acid metabolism in both outer and inner gall tissues suggests that the entire *D*. *quercuspalustris* gall supports transport of plant metabolites to the inner gall tissues via the connecting strands. Biochemical and histological studies have established that cells in the inner gall tissues closest to the feeding insect become specialized “nutritive” cells on which the insect feeds (Ferreira et al., 2019). Martinson et al. (2021) are the first to sample those tissues specifically for gene expression. They demonstrated elevated expression of genes encoding sucrose synthase in nutritive tissues, a suitably localised candidate mechanism for the strong sink effect of the inner gall. Expression of genes characteristic of gluconeogenesis was also greatly enhanced in the inner tissues. Martinson et al. (2021) suggest that glycolysis in these inner tissues might convert pyruvate, lactate or glycerol into glucose to feed the wasp larva. Starch often accumulates near the feeding insect in cynipid galls, but not in the cells actually consumed by the parasite. Interestingly, Martinson et al. (2021) found no differential expression of genes encoding starch degrading enzymes, suggesting that the wasp larva might digest starch on its own.

Since galling arthropods attack by chewing or sucking and siphoning resources away from their host, one might expect them to evoke defence responses. To the contrary, Martinson et al. (2021) found expression of defence‐related genes to be strikingly suppressed in the inner gall tissue and reduced in the outer gall. The few exceptions appear to be genes encoding proteins targeting microbes, especially fungi, which the authors point out are a major threat to galling insects. One possibility is thus that induced expression of these proteins is a defensive component of the gallwasp's extended phenotype. An alternative is that these proteins are defensive responses against eliciting or suppressing pathogens or gall‐inducing symbionts introduced by the galler. While symbionts are essential partners in some insect galls (e.g., fungal symbionts in galls induced by some cecidomyiid midges), neither Martinson et al. (2021) nor Hearn et al. (2019) found any evidence for symbiont involvement in cynipid galls. Martinson et al. (2021) also found upregulation of genes involved in phenylpropanoid synthesis in outer gall tissues, which could be defensive. However, phenylpropanoids are also involved in regulation of hormone transport and production of new cell walls in the growing gall. The annotations needed to reveal their functions in galls do not exist for *Q*. *rubra*. Some authors have tackled this problem by aligning sequences with those of a model plant, such as *Arabidopsis* (Schultz et al., 2019), which has its own shortcomings.

Martinson et al. (2021) sampled galls at a single time point in their development, so they cannot offer major insights about initiation of gall development or signals that control it. Phytohormones, some of which are produced by insects, are probably gall initiation and developmental signals (Tooker & Helms, 2014). While auxin signalling is central to plant organogenesis and has been implicated in development of other galls (Tooker & Helms, 2014), Martinson et al. (2021) found little evidence of it in this gall. This is probably because the influence of auxin often involves a few cells over a short time span (Galvan‐Ampudia et al., 2020). Like many others, Martinson et al. (2021) found upregulated expression of genes in ethylene synthesis and signalling pathways. However, ethylene's functions are many, including defence, growth, and development. The study by Martinson et al. (2021) is the second to report elevated expression of an ENOD gene in a cynipid gall (Hearn et al., 2019), which together with ethylene guides formation of gall‐like rhizobium nodules (Larrainzar et al., 2015). Martinson et al. (2021) also found elevated expression of genes involved in RNA modification, which may control expression of many developmental genes in the gall.

Molecular study of arthropod gall development is the only way we will understand how these remarkable structures come to be. To date, much of the functional inference from transcriptomic data has been based on gene ontology (GO) enrichment. However, this approach is not very informative without determining which genes are up‐ versus downregulated and what their impact may be in each case (Schultz et al., 2019). The study by Martinson et al. (2021) is one of few to go beyond simply listing enriched GO categories. Functional dissection of the roles of specific genes in gall induction and development is limited by a lack of galls formed on “model” plants (except for *Vitis* and *Populus*) with good functional annotation and the ability to modify the expression of key developmental genes over a time course (Betancourt et al., 2020). Relevant molecular and metabolomic resources are nevertheless growing rapidly, and genome editing technologies are increasingly applicable to nonmodel systems (Dickinson et al., 2020).

The study by Martinson et al. (2021) supports many observations made via histology, biochemistry, and a few transcriptome studies: the suppression of photosynthesis and development of a metabolic sink resulting in heterotrophy, suppressed defence responses close to the parasite, the metabolic characteristics of the tissues on which the parasite feeds, and the massive changes in gene expression compared with control tissues. They have, however, for the first time illustrated the spatial array of many of these changes on a transcriptional level. The picture that emerges is of a unique plant organ whose functional and anatomical complexity is matched and probably created by transcriptional changes controlled by the arthropod parasite. Exactly how the parasite achieves this remains unknown.

## References

[mec16482-bib-0001] Betancourt, E. K. , Soto, P. H. , Cortés, N. C. , Anaya, M. R. , Estrella, A. H. , & Oyama, K. (2020). Ecological genomics of plant‐insect interactions: the case of wasp‐induced galls. In J. Núñez‐Farfán & P. Valverde (Eds.), Evolutionary ecology of plant herbivore interaction (pp. 315–341). Springer.

[mec16482-bib-0002] Compson, Z. G. , Larson, K. C. , Zinkgraf, M. S. , & Whitham, T. G. (2011). A genetic basis for the manipulation of sink–source relationships by the galling aphid *Pemphigus betae* . Oecologia, 167, 711. 21667296 10.1007/s00442-011-2033-x

[mec16482-bib-0003] Dickinson, M. H. , Vosshall, L. B. , & Dow, J. A. T. (2020). Genome editing in non‐model organisms opens new horizons for comparative physiology. Journal of Experimental Biology, 223(Suppl 1), jeb221119. 10.1242/jeb.221119 32034052

[mec16482-bib-0004] Ferreira, B. G. , Álvarez, R. , Bragança, G. P. , Alvarenga, D. R. , Pérez‐Hidalgo, N. , & Isaias, R. M. S. (2019). Feeding and other gall facets: patterns and determinants in gall structure. Botanical Review, 85, 78–106. 10.1007/s12229-019-09207-w

[mec16482-bib-0005] Galvan‐Ampudia, C. S. , Cerutti, G. , Legrand, J. , Brunoud, G. , Martin‐Arevalillo, R. , Azais, R. , Bayle, V. , Moussu, S. , Wenzl, C. , Jaillais, Y. , Lohmann, J. U. , Godin, C. , & Vernoux, T. (2020). Temporal integration of auxin information for the regulation of patterning. eLife, 9, e55832. 10.7554/eLife.55832 32379043 PMC7205470

[mec16482-bib-0006] Giron, D. , Huguet, E. , Stone, G. N. , & Body, M. (2016). Insect‐induced effects on plants and possible effectors used by galling and leaf‐mining insects to manipulate their host‐plant. Journal of Insect Physiology, 84, 70–89. 10.1016/j.jinsphys.2015.12.009 26723843

[mec16482-bib-0007] Hearn, J. , Blaxter, M. , Schönrogge, K. , Nieves‐Aldrey, J.‐L. , Pujade‐Villar, J. , Huguet, E. , Drezen, J.‐M. , Shorthouse, J. D. , & Stone, G. N. (2019). Genomic dissection of an extended phenotype: Oak galling by a cynipid gall wasp. PLoS Genetics, 15, e1008398. 10.1371/journal.pgen.1008398 31682601 PMC6855507

[mec16482-bib-0008] Larrainzar, E. , Riely, B. K. , Kim, S. C. , Carrasquilla‐Garcia, N. , Yu, H. J. , Hwang, H. J. , Oh, M. , Kim, G. B. , Surendrarao, A. K. , Chasman, D. , Siahpirani, A. F. , Penmetsa, R. V. , Lee, G. S. , Kim, N. , Roy, S. , Mun, J. H. , & Cook, D. R. (2015). Deep sequencing of the *Medicago truncatula* root transcriptome reveals a massive and early interaction between nodulation factor and ethylene signals. Plant Physiology, 169, 233–265. 26175514 10.1104/pp.15.00350 PMC4577383

[mec16482-bib-0009] Martinson, E. O. , Werren, J. H. , & Egan, S. P. (2021). Tissue‐specific gene expression shows a cynipid wasp repurposes oak host gene networks to create a complex and novel parasite‐specific organ. Molecular Ecology, 31, 3228–3240. 10.1111/mec.16159 34510608

[mec16482-bib-0010] Nabity, P. D. , Haus, M. J. , Berenbaum, M. R. , & DeLucia, E. H. (2013). Leaf‐galling phylloxera on grapes reprograms host metabolism and morphology. Proceedings of the National Academy of Sciences of the USA, 110, 16663–16668. 10.1073/pnas.1220219110 24067657 PMC3799386

[mec16482-bib-0011] Schultz, J. C. , Edger, P. P. , Body, M. J. A. , & Appel, H. M. (2019). A galling insect activates plant reproductive programs during gall development. Scientific Reports, 9, 1833. 10.1038/s41598-018-38475-6 30755671 PMC6372598

[mec16482-bib-0013] Subramanyam, S. , Nemacheck, J. A. , Bernal‐Crespo, N. , & Sardesai, N. (2021). Insect derived extra oral GH32 plays a role in susceptibility of wheat to Hessian fly. Scientific Reports, 11, 2081. 10.1038/s41598-021-81481-4 33483565 PMC7822839

[mec16482-bib-0014] Tooker, J. F. , & Helms, A. M. (2014). Phytohormone dynamics associated with gall insects, and their potential role in the evolution of the gall‐inducing habit. Journal of Chemical Ecology, 40, 742–753. 10.1007/s10886-014-0457-6 25027764

